# HMGB1, angel or devil, in ischemic stroke

**DOI:** 10.1002/brb3.2987

**Published:** 2023-04-16

**Authors:** Bin Gao, Shuwen Wang, Jiangfeng Li, Nannan Han, Hanming Ge, Gejuan Zhang, Mingze Chang

**Affiliations:** ^1^ Department of Neurology Xi'an No. 3 Hospital the Affiliated Hospital of Northest University Xi'an Shaanxi P.R. China; ^2^ Department of Neurosurgery the First Hospital of Yu'lin Yu'lin Shaanxi China

**Keywords:** atherosclerosis, HMGB1, immune tissue repair, ischemic stroke, pathological mechanism

## Abstract

**Introduction:**

High‐mobility group box 1 protein (HMGB1) is extensively involved in causing ischemic stroke, pathological damage of ischemic brain injury, and neural tissue repair after ischemic brain injury. However, the precise role of HMGB1 in ischemic stroke remains to be elucidated.

**Methods:**

Comprehensive literature search and narrative review to summarize the current field of HMGB1 in cerebral ischemic based on the basic structure, structural modification, and functional roles of HMGB1 described in the literature.

**Results:**

Studies have exhibited the crucial roles of HMGB1 in cell death, immunity and inflammation, thrombosis, and remodeling and repair. HMGB1 released after cerebral infarction is extensively involved in the pathological injury process in the early stage of cerebral infarction, whereas it is involved in the promotion of brain tissue repair and remodeling in the late stage of cerebral infarction. HMGB1 plays a neurotrophic role in acute white matter stroke, whereas it causes sustained activation of inflammation and plays a damaging role in chronic white matter ischemia.

**Conclusions:**

HMGB1 plays a complex role in cerebral infarction, which is related to not only the modification of HMGB1 and bound receptors but also different stages and subtypes of cerebral infarction. future studies on HMGB1 should investigate the spatial and temporal dynamics of HMGB1 after cerebral infarction. Moreover, future studies on HMGB1 should attempt to integrate different stages and infarct subtypes of cerebral infarction.

## Graphical abstract

The topic combines one of the most death‐causing pathological condition with the reference DAMP protein, HMGB1, that has crucial roles in the pathophysiology of many inflammation‐related diseases. In this reviews,we explained its effect on different subtypes of cerebral infarction and its key mechanism based on the Spatiotemporal dynamics and Modifications of HMGB1 after cerebral infarction.

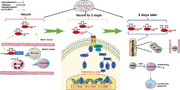

## 1 INTRODUCTION

Stroke is associated with the highest morbidity, mortality, and disability rates worldwide. In 2019, stroke was reported to be the third leading cause of death (Wang et al., 2022) and the leading cause of disability‐adjusted life‐year (DALY) in China (Ma et al., 2021). The current prevalence of stroke in Chinese adults aged ≥40 years has been reported to be 2.58% (Tu et al., 2022). In particular, the prevalence of ischemic stroke is extremely high. Patients with acute ischemic cerebrovascular disease are generally treated with intravenous thrombolysis or thrombectomy to restore blood reperfusion in the ischemic penumbra zone. However, these treatments are applicable only to a few patients due to strict indications and short‐time window for intravenous thrombolysis or thrombectomy. Recanalization of blood flow after ischemia can further lead to reperfusion injury. The activation of inflammatory cells after cerebral ischemia leads to the production of large amounts of proinflammatory mediators, with excessive release of excitatory amino acids and significant upregulation of intracellular Ca^2+^ and reactive oxygen species (ROS) in the neuronal cells. These processes interact with and promote each other, which cause severe damage to the neurons, glial cells, and endothelial cells and their interconnections, eventually leading to cellular damage and necrosis (Khoshnam et al., 2017). However, because of a lack of understanding of the cellular and molecular mechanisms following stroke, limited prophylactic and therapeutic measures are available for ischemic stroke to date.

High‐mobility group box 1 protein (HMGB1) is a member of the high‐mobility protein family and is a highly abundant nonhistone protein in the mammalian cell nuclei (Furuita et al., 2011; Lotze and Tracey, 2005; Mosevitsky et al., 1989). HMGB1 was first isolated from calf thymus chromatin non‐histone proteins in 1973 (Furuita et al., 2011) and was named so because of its high mobility in polyacrylamide gels and the nucleus (Stros, 2010). HMGB1, as classical damage‐associated molecular patterns (DAMPs), was initially studied in the context of immunity and inflammation. However, HMGB1 was also found to play vital roles in thrombosis, immunosuppression, and tissue repair.

The present study describes the role of HMGB1 in the complex pathophysiological mechanism of cerebral infarction and specifically analyses the role of HMGB1 in cerebral infarction subtypes to provide novel insights into the precise treatment of cerebral infarction.

## 2 HMGB1 DEFINITION AND STRUCTURE

**FIGURE 1:**
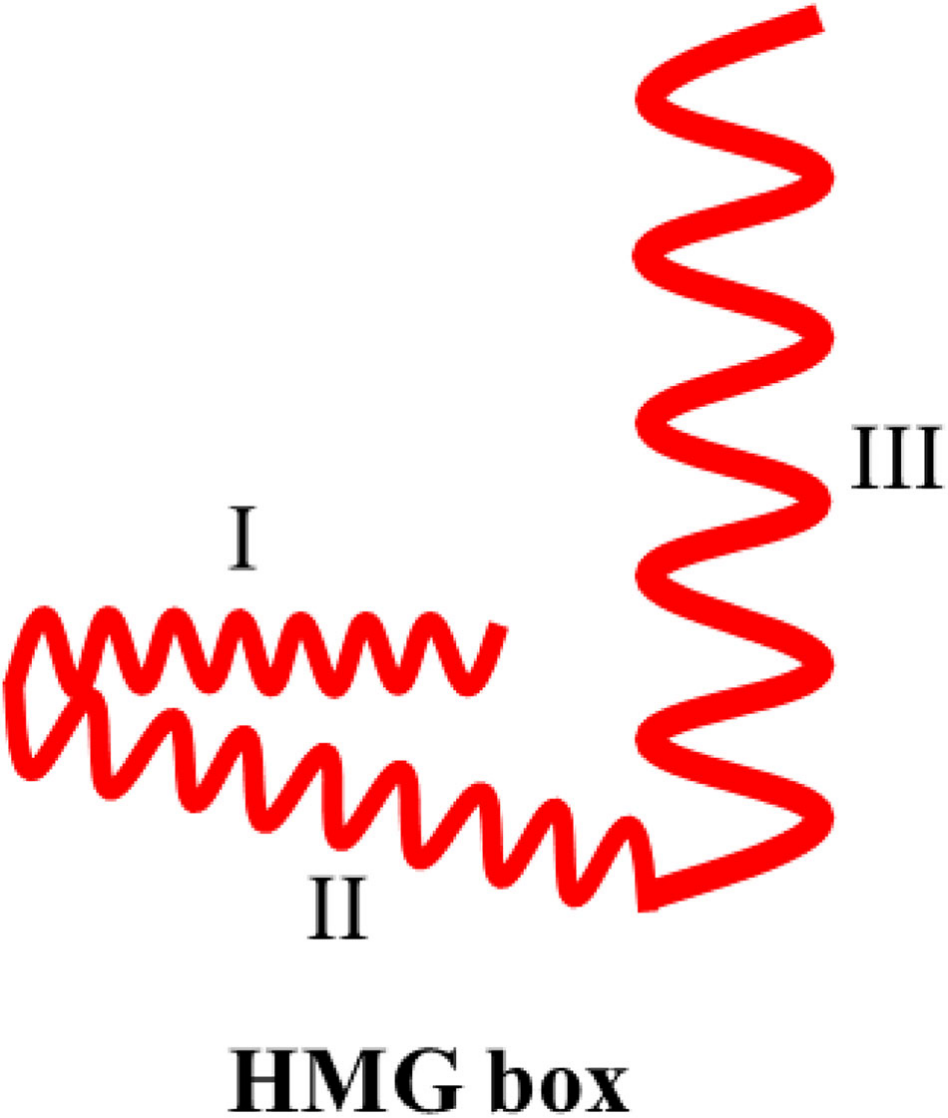
The structure of HMG box. Each HMG box comprises three ahelices arranged in a characteristic twisted ‘L’ structure, with helices I and II forming the short arm of the ‘L’ and helix III forming the long arm of the ‘L’. The angle between the two arms is approximately 80°.

**FIGURE 2:**
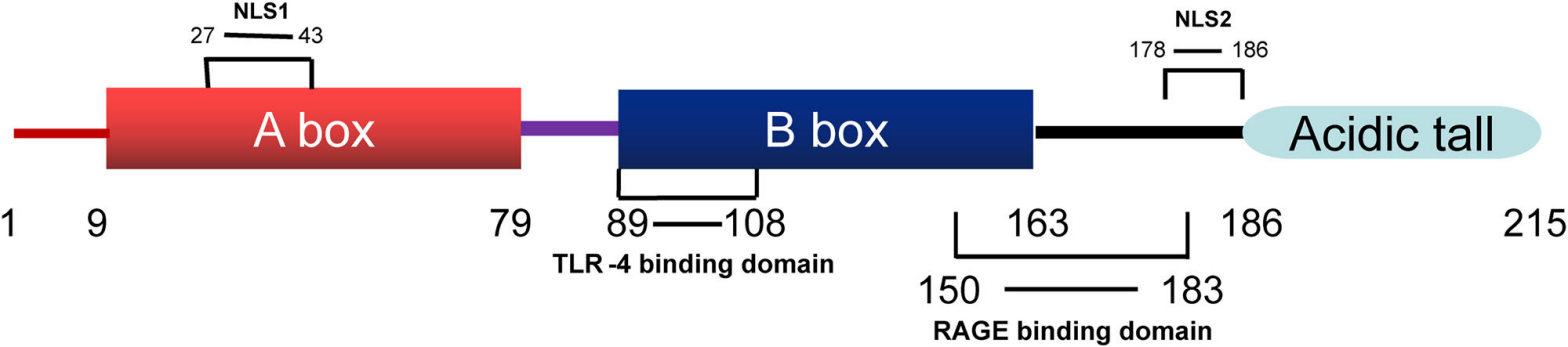
The structure and function of HMGB1. HMGB1 protein contains two DNA‐binding domains (HMG A box and HMG B box) and a C‐terminal tail. The HMG A box is a tertiary structure consisting of the amino acid residues 9‐79, whereas the HMG B box is a tertiary structure consisting of the amino acid residues 89‐163. The C‐terminal tail is composed of the amino acid residues 186‐215. The protein has three redox sites positioned at 23, 45, and 106 amino acid sequences for ‘SH ’‐ containing cysteine. NLS, including NLS1 and NLS2, plays a vitalrole in the nucleusctoplasm shuttling process of HMGB1. HMG B box contains the TLR‐4 binding site at the position 89‐ 108 of amino acid residuies, whereas the 150‐183 position contains the receptor binding site for RAGE.

Human HMGB1 comprises a peptide chain of 215 amino acid residues that forms a primary protein structure with a molecular weight of 25 KDa (Stros, 2010; Wang et al., 2019). HMGB1 exhibits 100% sequence homology between rats and mice and 99% sequence homology between humans and rodents (Andersson et al., 2002), indicating that HMGB1 may perform similar biological functions in different mammalian species. HMGB1 exhibits three structural domains, two positively charged DNA‐binding domains (HMG A box and HMG B box), and a negatively charged carboxy‐terminal “tail.” The HMG A box is a tertiary structure that comprises the amino acid residues 9−79, whereas the HMG B box is a tertiary structure that comprises the amino acid residues 89−163. These domains are interlinked by approximately 10 amino acid residues, followed by a carboxy‐terminal “tail” of 30 amino acid residues after the two DNA‐binding domain structures, which comprise the amino acid residues 186−215 interlinked with the HMG B cassette by approximately 23 amino acid residues (Cai et al., 2015; Musumeci et al., 2014; Tang et al., 2012). Each HMG box comprises three α‐helices arranged in a characteristic twisted “L” structure, with helices I and II forming the short arm of the “L” and helix III forming the long arm of the “L”; the angle between the two arms is approximately 80° (Bustin, 1999) (Figure 1; Hardman et al., 1995). Homology model construction experiments exhibited that the overall structural homology of the HMG box domain is more conserved than the amino acid sequence homology (Baxevanis et al., 1995). The HMGB1 protein peptide chain contains several functional signaling regions. For example, nuclear localization site 1 (NLS1) is located in the region consisting of amino acid residues 27−43, whereas NLS2 is located in the region consisting of amino acid residues 178−186 (Bonaldi et al., 2003). The binding site of receptor for advanced glycation end product (RAGE) consists of the amino acid residues 150−183 of the HMG B box (Huttunen et al., 2002), whereas that of the toll‐like receptors 4 (TLR‐4) consists of the amino acid residues 89−108 of the HMG B box (Harris et al., 2012) (Figure 2).

### 2.1 Posttranslational modifications of HMGB1

HMGB1 can be modified through redox, phosphorylation, methylation, and acetylation at different amino acid sites. The amino acid residues at positions 23, 45, and 106 of HMGB1 are the “‐SH”‐group containing cysteines. HMGB1 with “‐SH” in the cysteines at all three sites is called reduced‐HMGB1. When HMGB1 is mildly oxidized under the action of ROS, the “‐SH” groups of cysteines at positions 23 and 45 combine to form a disulfide bond, whereas the cysteine at position 106 still contains the “‐SH” group; this HMGB1 is called disulfide‐HMGB1. When “‐SH” groups at all three positions oxidize to form “‐SO_3_H”, the HMGB1 is called oxidized‐HMGB1 (Kohlstaedt et al., 1986; Yang et al., 2012). Among these forms, reversible redox reactions can occur between disulfide‐ and reduced‐HMGB1, whereas irreversible oxidation reactions occur when all three “‐SH” groups are oxidized (Venereau et al., 2012). Under physiological conditions, HMGB1 shuttles between the nucleus and cytoplasm, and the NLS plays a vital role in this process. HMGB1 loses its ability to shuttle into the nucleus in the monocytes when the lysine in the NLS is acetylated by the corresponding enzyme (probably histone acetylase) (Bonaldi et al., 2003). Studies have demonstrated the activation of the kinase/signal transducer and activator of transcription1 (JAK/STAT1) signaling pathway in monocytes upon stimulation by lipopolysaccharide (LPS) or type 1 interferon (INF‐1), which in turn causes the acetylation of intracellular HMGB1(Lu et al., 2014). Additionally, the stimulation of monocytes with tumor necrosis factor‐α (TNF‐α) and LPS‐induced serine phosphorylation around both NLS regions has been reported, with the phosphorylated HMGB1 incapable of entering the nucleus (Oh et al., 2009; Youn and Shin, 2006). The mechanism through which serine phosphorylation occurs is determined by the phosphoinositide 3‐kinase/phosphoinositide‐dependent kinase‐1 (PI3K/PDK1) signaling pathway that activates conventional protein kinase C (cPKC), which results in the translocation of cPKC into the nucleus in a Ca^2+^‐dependent mechanism of serine phosphorylation in the NLS region (Oh et al., 2009). The methylated HMGB1 exhibits reduced affinity to chromosomal DNA upon methylation of lysine at position 42 of HMGB1 in neutrophils and thus diffuses passively from the nucleus to the cytoplasm (Ito et al., 2007). However, the specific mechanism of HMGB1 methylation remains to be investigated further.

### 2.2 HMGB1 release

Intracellular HMGB1 is released into the extracellular space through two major routes, namely active secretion and passive release. Active HMGB1 secretion from the inflammatory cells into the extracellular space generally occurs in two steps. In the first step, posttranslational modification of HMGB1 occurs in response to various inflammatory or pathogenic microbial stimuli, which leads to HMGB1 aggregation in the cytoplasm. In the second step, cytoplasmic HMGB1 cannot be released into the extracellular space by the endoplasmic reticulum–Golgi pathway due to the lack of a precursor peptide, and HMGB1 aggregates in the cytoplasm are released into the extracellular space by secreted lysosomes (Bonaldi et al., 2003; Tang et al., 2016). IFN‐1 or LPS stimulates inflammatory cells to activate the JAK/STAT1 signaling pathway, which mediates HMGB1 acetylation, causing HMGB1 to aggregate in the cytoplasm (Lu et al., 2014). Interferon regulatory factor 1 (IRF‐1) is a downstream factor of the JAK/STAT1 signaling pathway in macrophages, and its activation promotes HMGB1 acetylation by histone acetyltransferases (Dhupar et al., 2011; Rendon‐Mitchell et al., 2003). However, rapamycin and H_2_O_2_ that stimulate HMGB1 aggregation in the cytoplasm and its accumulation in the inflammatory cells are not associated with the JAK/STAT1 signaling pathway (Lu et al., 2014), suggesting that different stimuli induce distinct signaling pathways for HMGB1 cytoplasmic aggregation. Furthermore, HMGB1 cytoplasmic aggregation within the same cell occurs through multiple signaling pathways. For example, HMGB1 in the monocytes can undergo cytoplasmic aggregation through both acetylation and phosphorylation modifications (Bonaldi et al., 2003; Youn and Shin, 2006), whereas HMGB1 in the neutrophils can undergo cytoplasmic aggregation only through methylation (Ito et al., 2007), suggesting that the signaling pathway of HMGB1 release may also vary across different cell types. The signaling pathways for HMGB1 release differ with cell death modalities. Pyroptosis is the process of programmed death of immune cells (Lamkanfi et al., 2010). Monocytes can undergo pyroptosis in response to LPS or INF stimulation, and HMGB1 released by pyroptosis is generally highly acetylated modified reduced‐HMGB1 or disulfide‐HMGB1 (Hornung et al., 2009; Jin et al., 2012). Assembly and activation of inflammatory vesicles, which are crucial for the occurrence of pyroptosis, can activate caspase1/caspase11 in the process (Kayagaki et al., 2011; Lamkanfi et al., 2010; Willingham et al., 2009), eventually mediating HMGB1 release from the cytoplasm into the extracellular space (Kayagaki et al., 2011; Willingham et al., 2009). However, further investigation is required to understand the mechanism of HMGB1 release into the extracellular space during pyroptosis. Apoptosis refers to programmed cell death and is marked formatively by cell crumpling, chromosome condensation, and apoptotic vesicle morphology. Cells generally release oxidized‐HMGB1 during apoptosis, and oxidized‐HMGB1 released by apoptotic cells is vital for inducing immune tolerance (Kazama et al., 2008). However, if apoptotic body degradation is insufficient, HMGB1–nucleosome complexes form in the late apoptotic stages that bind to TLR2 to exert proinflammatory effects (Urbonaviciute et al., 2008). Caspase‐activated DNase (CAD) and DNase γ play a role in the cleavage of genomic DNA during apoptosis, and it was found that the process of apoptosis by CAD and DNase γ catalyzed nuclear DNA fragmentation plays a key role in the release of HMGB1 during apoptosis (Yamada et al., 2011). HMGB1 is passively released from the cells when necrosis occurs, and necrotic cells usually release reduced‐HMGB1 without acetylation modification (Scaffidi et al., 2002; Tang et al., 2016). A few studies have demonstrated the activation of poly(ADP)‐ribose polymerase (PARP‐1) by DNA in cells after alkylation damage, and the activated PARP‐1 further mediates cellular necrosis. Additionally, PARP‐1 promotes the cytoplasmic aggregation process of HMGB1 prior to the onset of cellular necrosis. During this process, the plasma membrane cleaves, and HMGB1 aggregates in the cytoplasm are released from the cell (Ditsworth et al., 2007). Cellular autophagy is a process through which the cells degrade intracellular materials by removing misfolded proteins and damaged organelles and by invading microorganisms to maintain cellular homeostasis (Levine and Kroemer, 2019). Glioblastoma treatment with the anticancer drug epidermal growth factor receptor‐targeted diphtheria toxin (DT‐ EGF) induces autophagy and HMGB1 release from tumor cells, and HMGB1 release is greatly reduced with autophagy inhibition. Activation of autophagy prior to DT‐EGF treatment also induces HMGB1 release, suggesting that cellular autophagy can induce HMGB1 release (Thorburn et al., 2009). A study exhibited the induction of HMGB1 release by H_2_O_2_ through mitogen‐activated protein kinase (MAPK) and chromosome region maintenance 1 (CRM1)‐dependent mechanisms to promote HMGB1 release from the monocytes (Tang et al., 2007). Under hypoxia, the human nasal mucosal epithelial cells release HMGB1 in a ROS‐dependent manner (Min et al., 2017), suggesting that oxidative stress promotes HMGB1 cellular secretion. Studies have exhibited that “‐SH” oxidation at position 106 cysteine drives HMGB1 accumulation in the cytoplasm (Hoppe et al., 2006), which represents one of the plausible mechanisms by which oxidative stress induces HMGB1 translocation into the cytoplasm. However, the mechanism of oxidative stress‐mediated HMGB1 release from the cytoplasm into the extracellular space remains unclear.

### 2.3 HMGB1 features

**FIGURE 3:**
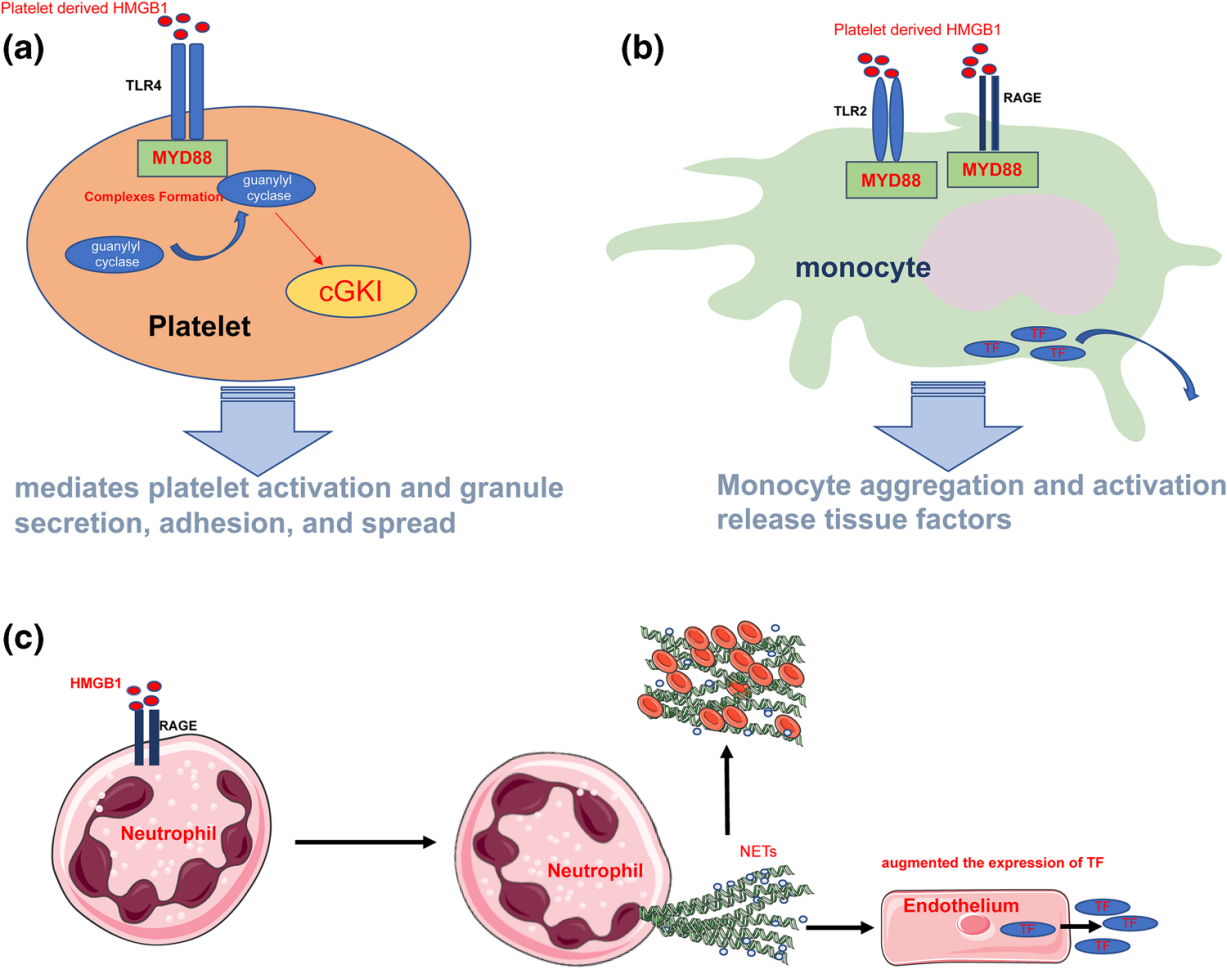
HMGB1 promotes thrombus formation. (a) HMGB1 acts on TLR4 on platelet membranes, causing the recruitment of guanylyl cyclase (GC) to the platelet membranes through the TLR4/myeloid differentiation factor88 (MyD88) pathway, followed by the formation of MyD88/GC complexes. This process further activates cyclic guanosine monophosphate (cGMP)‐dependent protein kinase l (cGKI), which mediates platelet activation and granule secretion, adhesion, and spread. (b) HMGB1 acts on RAGE and TLR2 on the monocyte membrane surface to promote monocyte aggregation. Monocyte aggregation and activation release tissue factors. (c) HMGB1 acts on RAGE on neutrophil membrane surfaces to promote autophagy in neutrophils, which increases the formation of neutrophil extracellular traps (NETs). NETs Provide a Scaffold and Stimulus for Platelet Binding and Aggregation, besides promote endothelial cells to express TF.

A study reported death of HMGB1‐knockout mice from hypoglycemia 24 h after their birth, and the mice exhibited defects in the glucocorticoid receptor transcriptional function, which highlights the life‐supporting function of HMGB1 (Calogero et al., 1999). The function of HMGB1 depends on its location in the cells, its posttranslational modifications, and the receptors it binds. HMGB1 loosens wrapped DNA in the nucleus and enhances chromatin remodeling complexes and accessibility of transcription factors. Additionally, HMGB1 enhances the affinity of many sequence‐specific transcription factors (estrogen receptor, p53, and p73) to DNA and promotes transcription (Bianchi and Agresti, 2005). Furthermore, nuclear HMGB1 is involved in DNA replication, repair (Mandke and Vasquez, 2019), chromatin stability, and chromatin remodeling (Bustin and Reeves, 1996; Bustin et al., 1990; Stros et al., 2007), whereas cytoplasmic HMGB1 is involved in cellular autophagy under stress conditions. Cellular ROS production increases in cells, and ROS oxidizes the “‐SH” of cysteine at position 106 of HMGB1, which promotes HMGB1 cytoplasmic aggregation. This HMGB1 aggregate disrupts the interaction between beclin1 and Bcl‐2 by binding competitively with beclin1, thereby inhibiting the apoptotic process under stress conditions and promoting cellular autophagic survival (Kang et al., 2011; Tang et al., 2010). Cytoplasmic HMGB1 regulates the phagocytic capacity of macrophages. HMGB1 interacts with Src kinase and inhibits Src binding to focal adhesion kinase (FAK), thereby inhibiting FAK phosphorylation and activation and reducing the ability of macrophages to phagocytose the apoptotic cells (Banerjee et al., 2011). HMGB1 is distributed in the cell membranes, particularly neuronal cell membranes and platelet membranes, and extracellular spaces. HMGB1 promotes thrombus formation (Rouhiainen et al., 2000) through several mechanisms. First, HMGB1 acts on TLR4 in platelet membranes, causing the recruitment of guanylyl cyclase (GC) to the platelet membranes through the TLR4/myeloid differentiation factor 88 (MyD88) pathway, followed by the formation of MyD88/GC complexes. This process further activates cyclic guanosine monophosphate (cGMP)‐dependent protein kinase I (cGKI), which mediates platelet activation and granule secretion, adhesion, and spread (Vogel et al., 2015) (Figure 3a). Second, HMGB1 acts on RAGE and TLR2 on the monocyte membrane surface to promote monocyte aggregation. Monocyte aggregation and activation release tissue factors (Stark et al., 2016) (Figure 3b). Third, HMGB1 acts on RAGE on neutrophil membrane surfaces to promote autophagy in neutrophils, which increases the formation of neutrophil extracellular traps (NETs) (Maugeri et al., 2014). NETs promote platelet aggregation and clotting activation, whereas platelet aggregation and NET formation promote HMGB1 release. This positive feedback process promotes thrombus formation and enlargement (Stark et al., 2016) (Figure 3c). Disulfide‐HMGB1 promotes thrombus formation and enlargement (Stark et al., 2016); however, reduced‐HMGB1 and oxidized‐HMGB1 are not involved in this process. HMGB1, as classical DAMP, can activate the immune system and exert proinflammatory effects. C3a complement activation products and C5b‐9 tapping complexes on the cell membranes surfaces can be detected after the addition of HMGB1 to human plasma, suggesting that HMGB1 can activate the complement system. Furthermore, studies have exhibited that reduced‐HMGB1, disulfide‐HMGB1, and oxidized‐HMGB1 can all bind to C1q and activate the complement system through a nonclassical pathway (Kim et al., 2018). HMGB1 can also form complexes with endogenous or exogenous proinflammatory molecules such as interleukin‐1α (IL‐1α), interleukin‐1β (IL‐1β) (Sha et al., 2008), stromal cell‐derived factor‐1 (SDF‐1) (Campana et al., 2009), nucleosomes (Urbonaviciute et al., 2008), LPS (Hreggvidsdottir et al., 2009), and CpG (Hreggvidsdottir et al., 2009). HMGB1 binds to different proinflammatory molecules to form complexes that enhance the proinflammatory function of these molecules, forming complexes with molecules such as IL‐1α and IL‐1β. By increasing the local concentration of chaperone molecules such as IL‐1α and IL‐1β and promoting their binding to receptors to stimulate signaling, the HMGB1 bound to these proinflammatory molecules can be reduced or oxidized (Hreggvidsdóttir et al., 2012) and binds to CpG oligodeoxynucleotides (CpG‐ODN) to form a complex that promotes CpG‐ODN endocytosis by interacting with RAGE. HMGB1 promotes the binding of CpG‐ODN to intracellular TLR‐9 and enhances the proinflammatory capacity of CpG‐ODN (Ivanov et al., 2007; Tian et al., 2007). HMGB1 peptide chains have a binding site for LPS in each of the A and B boxes (Youn et al., 2011). HMGB1 binds to LPS to form a complex through RAGE internalized into the lysosomes. HMGB1 permeates the phospholipid bilayer in the acidic environment of the lysosome, which leads to LPS leakage into the cytosol and caspase‐11 activation, resulting in pyroptosis. This process causes the release of several proinflammatory mediators into the extracellular environment (Deng et al., 2018). Reduced‐HMGB1 can form a heterodimer with C‐X‐C motif chemokine 12 (CXCL12), which enhances CXCL12 binding to C‐X‐C chemokine receptor type 4 (CXCR4) and promotes leucocyte migration (Schiraldi et al., 2012). Additionally, disulfide‐HMGB1 induces cytokine release, where the “‐SH” group on cysteine at position 106 is essential for HMGB1 to perform its procytokine release function. Disulfide‐HMGB1 uses the sequence around Cys106 to recognize and bind with the TLR4 coreceptor myeloid differentiation factor‐2 (MD‐2) and promote nuclear transcription factor‐κB (NF‐κB) activation upon binding to TLR4‐MD‐2 through the MyD88 pathway, leading to the subsequent release of TNF‐α, IL‐1, and IL‐6. MD‐2 exhibits 1000‐fold greater affinity for disulfide‐HMGB1 than for other HMGB1 isoforms (reduced‐HMGB1 and oxidized‐HMGB1), ensuring that only disulfide‐HMGB1 can bind to TLR‐4 and perform cytokine‐promoting functions (Yang et al., 2015). HMGB1 binds to RAGE on the neutrophil membrane surface and promotes neutrophils migration by facilitating the interaction of RAGE with membrane surface β2‐integrin macrophage‐1 (Mac‐1) (Orlova et al., 2007). It also promotes dendritic cell (DC) maturation, and matured DCs can migrate to secondary lymphoid tissues. Additionally, HMGB1 promotes T‐cell activation and polarization to Th1 cells by promoting T‐cell costimulatory protein molecule expression on the DC membrane surface and IL‐12 secretion by DCs (Dumitriu et al., 2005). Cell phagocytosis is crucial for the dissipation of inflammation. HMGB1 interacts with phosphatidylserine (PS) on the membrane surface of apoptotic neutrophils, thereby inhibiting phagocytosis of apoptotic neutrophils and promoting continued activation of the immune system (Liu et al., 2008). However, HMGB1 not only performs immune activation and proinflammatory functions but also exerts immunosuppressive effects. Oxidized‐HMGB1 exhibits immune unresponsive properties. Apoptotic cells promote HMGB1 oxidation due to the massive production of intracellular ROS, which eventually releases oxidized‐HMGB1 that is involved in the induction of immune tolerance by apoptotic cells in the process of immune tolerance (Kazama et al., 2008). Although HMGB1 activates macrophages, it causes apoptosis of macrophages in concentration‐ and time‐dependent manners through RAGE (Zhu et al., 2009). HMGB1 promotes the differentiation of myeloid‐derived suppressor cells (MDSCs) and contributes to the suppression of CD4^+^ and CD8^+^ T‐cell activation. Additionally, HMGB1 activates MDSC through the NF‐κB signaling pathway, promoting MDSC secretion of IL‐10 and helping MDSCs in downregulating naive T‐cell L‐selectin expression, thereby inhibiting the homing of naive T cells to lymph nodes (Parker et al., 2014). HMGB1 polarized CD4^+^ T cells toward Th1 through DCs. However, the effect of HMGB1 on CD4^+^ T cells is correlated with its concentration and duration of action. Small HMGB1 doses polarize CD4^+^ T cells toward Th1, whereas high HMGB1 doses and prolonged stimulation polarize them toward Th2 (Zhao et al., 2012). Additionally, high HMGB1 doses induce the apoptosis of T cells through the mitochondrial apoptotic pathway, which directly disrupts the adaptive immune response and indirectly induces the generation of immune tolerance in macrophages and DCs that phagocytose these apoptotic cells (Wu et al., 2014). Treg cells maintain immune tolerance and suppress inflammation. Compared with conventional T cells, the distribution of RAGE on the surface of Treg cells in HMGB1 induces Treg cell migration, promotes Treg cell survival, and enhances their immunosuppression by binding to Treg cell surface RAGE to promote IL‐10 secretion (Wild et al., 2012). HMGB1 interferes with the functional assembly of NADPH oxidase (NOX) by inhibiting p40phox subunit phosphorylation, whereas the interaction between HMGB1 and RAGE effectively inhibits NOX activation in neutrophils.(Tadié et al.,^2012^)In septic mice and patients with infectious shock, long‐term accumulation of extracellular HMGB1 promotes the development of NOX dysfunction and immunosuppression with neutrophils (Grégoire et al., 2017). GB1 in the early stages of inflammation can promote monocyte migration and activation. However, when HMGB1 binds to C1q, it catalyzes the formation of a multimeric protein complex of HMGB1, C1q, leukocyte‐associated immunoglobulin‐like receptor‐1 (LAIR‐1), and RAGE, which triggers monocytes to acquire an anti‐inflammatory (M2) phenotype, leading to upregulation of the expression of anti‐inflammatory molecules such as CD163, programmed death‐ligand 1 (PD‐L1), Mer tyrosine kinase (Mer), and IL‐10. The interaction between C1q and HMGB1 correlates with their relative concentrations. At low serum HMGB1 concentrations, C1q inhibits the proinflammatory capacity of HMGB1, whereas at high concentrations, C1q converts the macrophage phenotype from proinflammatory (M1 phenotype) to anti‐inflammatory (M2 phenotype). At extremely high HMGB1 concentrations, C1q fails to inhibit the proinflammatory effects of HMGB1 (Son et al., 2016). Extracellular HMGB1 exhibits a repair function, in addition to its prothrombotic, proinflammatory, and anti‐inflammatory functions. HMGB1 released from damaged tissues recruits mesenchymal stem cells, which play a crucial role in the repair of connective tissues such as bones, cartilages, muscles, bone marrow stroma, and tendons (Bianchi et al., 2017). The role of HMGB1 in promoting vascular regeneration in ischemic areas has been extensively investigated. HMGB1 induces endothelial progenitor cell (EPC) chemotactic migration in RAGE‐ and integrin‐dependent manners by binding to RAGE on the EPC membrane surfaces. HMGB1 promotes EPC adhesion to mature endothelial cells and fibronectin, as well as accelerates EPC migration to ischemic tissues (Chavakis et al., 2007). Additionally, HMGB1 regulates the synthesis of fibronectin and the γ2‐chain of laminin‐5 through RAGE and TLR‐4, which are involved in bronchial epithelial repair after injury (Ojo et al., 2015). The CXCL12/CXCR4 axis is crucial for HMGB1‐mediated repair after muscle injury. After injury, reduced‐HMGB1 binds to CXCL12 to form a complex that promotes the migration of myogenic cells by acting on CXCR4 receptors, whereas HMGB1 promotes the repair of injured muscle tissues by promoting myogenic cell activation and proliferation, and construction of a tissue‐healing microenvironment (Tirone et al., 2018). GAlert stem cells represent a state that is intermediate between the G0 and G1 stem cell states. In the presence of activators, GAlert stem cells can enter the cell cycle faster than quiescent stem cells, thereby accelerating tissue repair. Reduced‐HMGB1 promotes the conversion of quiescent stem cells from G0 to GAlert through the CXCL12/CXCR4 axis, thereby accelerating the healing of injured tissues (Lee et al., 2018). Additionally, HMGB1 is involved in the whole process of inflammation, thereby recruiting inflammatory cells in the early stage of inflammation, promoting the onset and development of inflammation, promoting the dissipation of inflammation in the late stage of inflammation, and promoting the repair of damaged tissues (Table 1).

**TABLE 1:** Functions and mechanisms of HMGB1

Location	Function	Mechanism
**Intranuclear**	Transcription	① Loose DNA (Bianchi and Agresti, 2005) ② Enhancement of transcription factor affinity with DNA Loose DNA (Bianchi and Agresti, 2005)
	DNA repair	① Promotes recognition of damaged DNA (Mandke and Vasquez, 2019) ② Promotes the repair of DNA repair enzymes (Mandke and Vasquez, 2019)
	Chromatin stability	Promotes the repairing effect of telomerase (Mandke and Vasquez, 2019)
	Chromatin remodeling	Nucleosome sliding (Mandke and Vasquez, 2019)
**Cytoplasm**	Promotes autophagy	Disrupts the interaction between beclin1 and Bcl‐2 (Kang et al., 2011; Tang et al., 2010)
	Inhibits phagocytosis	Inhibits the binding of Src to FAK, thereby inhibiting FAK phosphorylation and activation (Banerjee et al., 2011)
**Extracellular extracellular**	Promotes thrombosis	① TLR4/MyD88/GC/cGKI signaling pathway activation (Vogel et al., 2015) ② Promotes monocyte–platelet complex formation through the RAGE/TLR‐2 pathway (Vogel et al., 2015) ③ Platelet aggregation and coagulation activation were promoted by facilitating NET formation (Maugeri et al., 2014)
	Promotes inflammation and immune activation	① Binds to C1q and activates the complement system (Kim et al., 2018) ② Forms complexes with endogenous or exogenous proinflammatory molecules, enhancing the proinflammatory function of these molecules (Kang et al., 2011; Tang et al., 2010) ③ Promotes leucocyte migration through the CXCL12/CXCR4 pathway (Schiraldi et al., 2012) ④ Inhibits phagocytosis of apoptotic neutrophils by phagocytes (Liu et al., 2008)
	Suppresses inflammation and causes immunosuppression	① Oxidized‐HMGB1 is involved in the induction of immune tolerance by apoptotic cells (Kazama et al., 2008) ② Promotes apoptosis of macrophages through RAGE (Kazama et al., 2008) ③ Promotes MDSC production (Parker et al., 2014) ④ Promotes Th2 cell and M2‐type cell production (Kang et al., 2011; Tang et al., 2010) ⑤ Promotes Treg cell migration and survival (Wild et al., 2012)
	Tissue repair	① Promotes EPC migration and adhesion (Chavakis et al., 2007) ② Promotes myosatellite cell migration through the CXCL12/CXCR4 axis pathway (Tirone et al., 2018) ③ Promotes stem cell transformation from G0 to _GAlert_ through the CXCL12/CXCR4 pathway (Lee et al., 2018)

### 2.4 Role of HMGB1 in atherosclerosis

Atherosclerosis is characterized by the appearance of atheromatous materials, which are formed by lipid deposition and necrosis in the intima of large‐ and medium‐sized arteries, as well as by the proliferation of smooth muscle cells and fibrous tissues. Chronic inflammatory response is crucial in atherosclerosis development and progression. The formation and developmental stages of atherosclerotic plaques are accompanied by an increase in the HMGB1 content. Studies have exhibited that although smooth muscle and endothelial cells release HMGB1, macrophages are the main source of HMGB1 in atherosclerotic plaques (Kalinina et al., 2004; Porto et al., 2006). Extracellular HMGB1 release induces the release of large amounts of inflammatory factors such as TNF‐α, IL‐1, and IL‐6 from monocytes. Endothelial cells expresses HMGB1 by promoting the expressions of intercellular cell adhesion molecule‐1 (ICAM‐1), vascular cell adhesion molecule 1 (VCAM1), and RAGE, thereby accelerating inflammatory cell infiltration (Kalinina et al., 2004). Additionally, HMGB1 promotes atherosclerosis development and progression by promoting smooth muscle cell migration and proliferation (Porto et al., 2006). A study reported that it reduced atherosclerosis by 55%; decreased the infiltration of inflammatory cells such as T cells and monocytes; reduced the levels of inflammatory mediators such as IL‐1, TNF‐α, and monocyte chemotactic protein‐1 (MCP‐1); and reduced the inflammatory response within the plaque (Kanellakis et al., 2011). Vulnerable plaques are the major cause of ischemic lesions in vital organs due to atherosclerosis, and HMGB1 also plays a vital role in plaque vulnerability. Studies have exhibited the upregulation of matrix metalloproteinase (MMP) expression by HMGB1 (Kohno et al., 2012; Qiu et al., 2010). This process is vital for atherosclerotic plaque rupture (Lee and Libby, 1997). A study exhibited that low‐density lipoprotein (LDL) stimulates the translocation of HMGB1 from the cytoplasm to the nucleus in endothelial cells. Additionally, the study found that HMGB1 translocated to the nucleus by stabilizing the transcription factor sterol regulatory element‐binding protein 2 (SREBP2) of scavenger receptor B1 (SR‐B1), promoting SR‐B1 expression in endothelial cells, which mediates LDL translocation across endothelial cells and LDL accumulation in the endothelium (Zhang and Fernández‐Hernando, 2021). The effect of HMGB1 on atherosclerosis may also be related to its redox status. A study exhibited that pluripotent vascular stem cells proliferate and differentiate into smooth muscle cells in response to the platelet‐derived growth factor that accelerates atherosclerosis. Reduced‐HMGB1 can inhibit pluripotent vascular stem cell proliferation and differentiation, whereas oxidized‐HMGB1 loses its ability to inhibit the proliferation and differentiation of these cells, indicating that HMGB1 with different redox states may regulate atherosclerosis (Meng et al., 2018).

## 3 MODIFICATIONS AND RELEASE OF HMGB1 AFTER CEREBRAL INFARCTION

Extracellular transfer of HMGB1, following ischemia, is a time‐dependent and cell type‐specific process. After ischemic stroke onset, the neuronal cells that undergo necrosis in the early infarct core rapidly and abundantly release nonacetylated hmgb1, which peaks 24 h after infarction (Kim et al., 2006, 2008). Additionally, damps such as S100B and ATP are released into the extracellular space during ischemia and in large quantities in the early phase of ischemia. The released damps activate microglia and peripheral immune cells, which continually release HMGB1 (An et al., 2014). Ischemic penumbra region, microglia, astrocytes, and microvascular endothelial cells slowly release modified HMGB1 (Kim et al., 2008), mostly in the acetylated form, under ischemic stimulation. These acetylated hmgb1 actively secreted by the immune cells reach a peak approximately at day 6 after cerebral infarction (Kim et al., 2006). In a mouse model of middle cerebral artery occlusion (MCAO), HMGB1 was present predominantly in the form of reduced‐HMGB1 in serum, with the level of disulfide‐HMGB1 being extremely low at 2 h after cerebral infarction; however, a gradual increase in the serum disulfide‐HMGB1 concentration was observed with an increase in the infarction duration (Liesz et al., 2015). Liesz et al. (2015) performed specific HMGB1 staining in ischemia‐induced necrotic neurons and astrocytes by using hmgb1 antibody and observed that the cytoplasmic HMGB1 staining was almost uniform in the necrotic neurons, whereas it was granular in the necrotic astrocytes, which indicated that the posttranslational hmgb1 modifications in ischemia‐induced neurons, glial cells, and the secretory pathway may vary. HMGB1 in the ischemia‐induced neurons is transferred from the nucleus to the cytosol and protrusions, and it is further released into the extracellular space through secretory lysosomes (Faraco et al., 2007; wu et al., 2018). In these processes, the JAK2/STAT3 signaling pathway plays a crucial role. Inhibition of the JAK2/STAT3 signaling pathway by using curcumin can reduce HMGB1 expression after cerebral ischemia (Wu et al., 2018). However, the mechanism of active HMGB1 secretion after cerebral infarction is poorly understood

### 1 HMGB1 and excitatory amino acid toxicity

In response to ischemic stimuli, the release of glutamate‐based excitatory amino acids from the intracellular compartment into the extracellular compartment leads to an increase in glutamate levels in the extracellular compartment. The underlying mechanism mainly involves the release of glutamate from the cystine/glutamate retrotransporter, as well as the dysfunction or even retro‐transportation of the glutamate uptake system, which mainly comprises excitatory amino acid transporters (EAAT) [e.g., glutamate transporter‐1 (GLT‐1) and glutamate aspartate transporter (GLAST)] (Soria et al., 2014). In a mouse model of transient MCAO, glutamate concentrations increased rapidly at the onset of cerebral ischemia, reduced during reperfusion, and increased again 1 h after reperfusion; however, anti‐HMGB1 antibody could prevent the increase in glutamate concentration after reperfusion (Zhang et al., 2011), suggesting that HMGB1 promotes extracellular glutamate aggregation after cerebral ischemia. Lin et al. (2020) observed that a large amount of released HMGB1 downregulates GLAST through TLR4 expression after cerebral ischemia, thereby inhibiting astrocyte glutamate clearance. Thus, HMGB1 released after ischemic stroke promotes excitatory amino acid toxic effects.

### 2 HMGB1 and oxidative/nitrification stress

ONOO, an oxidant/nitrator that is produced after cerebral infarction, exerts stronger cytotoxic effects than O^2–^ and nitric oxide (NO) (Suzuki et al., 2002). Chen et al. (2020) reported that the elevated plasma HMGB1 levels in patients with cerebral infarction may be related to ONOO production. Furthermore, in the MCAO mouse model, ONOO was found to promote HMGB1 and TLR‐2 expressions and activate the HMGB1/TLR‐2 signaling pathway, which in turn promoted MMP9 expression and eventually the hemorrhagic transformation. Oxidation of amino acid residues at position 106 may be the mechanism through which oxidative stress promotes extracellular HMGB1 release (Hoppe et al., 2006). However, Chen et al. (2020) observed that HMGB1 induced by ONOO can act on TLR‐2, suggesting that ONOO‐induced HMGB1 release may occur independently of the mechanism of amino acid residue oxidation at position 106.

### 3 HMGB1 and immunity after cerebral infarction

**FIGURE 4:**
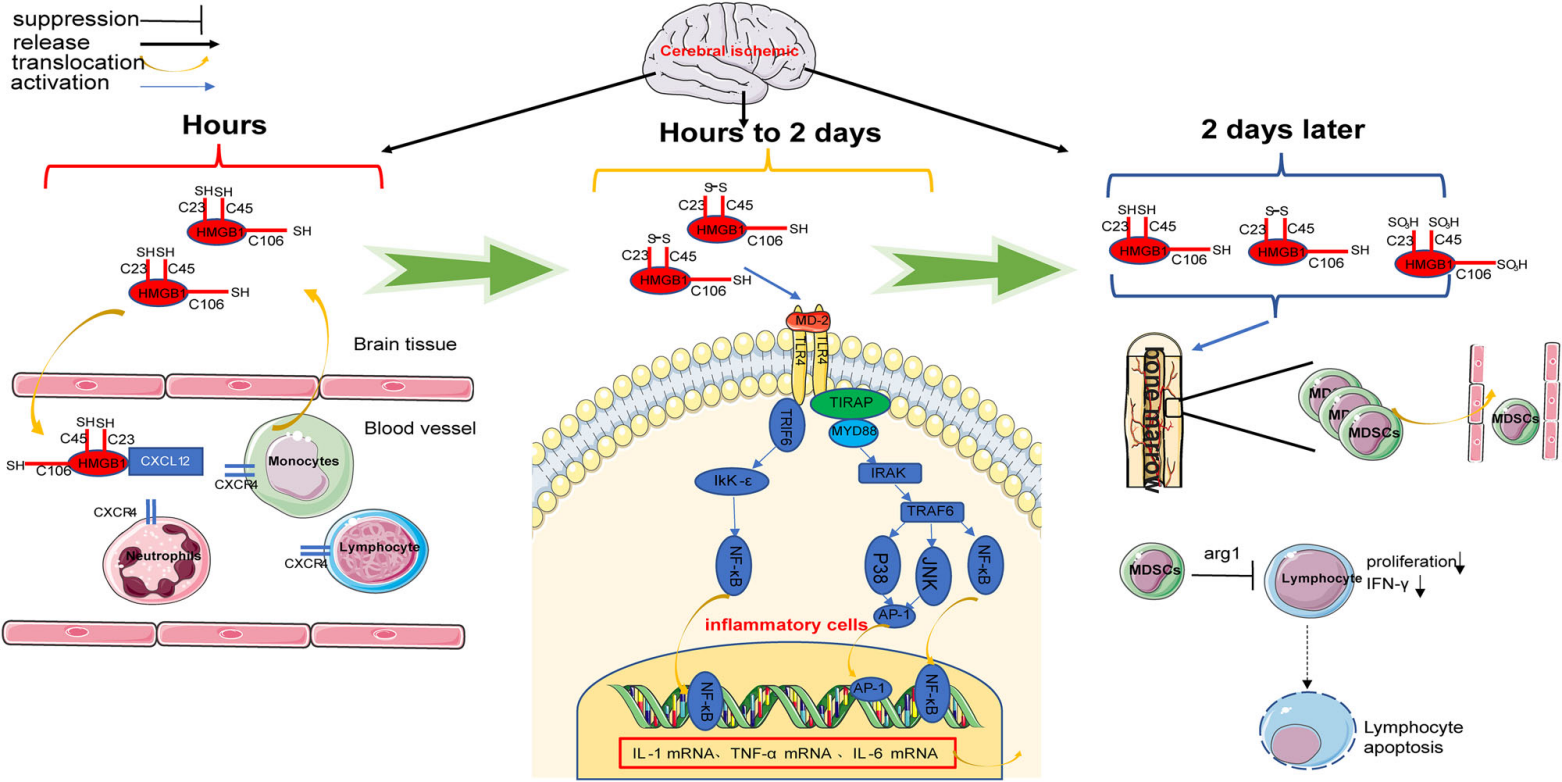
HMGB1 and immunity after cerebral infarction.After ischaemic stroke onset, the neuronal cells rapidly and abundantly release reduced‐HMGB1 within hours.and Reduced‐HMGB1 forms a heterodimer with CXCL12, which enhances CXCL12 binding to CXCR4 and promotes leucocyte migration.Disulfide‐HMGB1 increases gradually 24 h after infarction, Disulfide‐HMGB1 recognise and bind with the TLR4 co‐receptor myeloid differentiation factor‐2 (MD‐2) and promote nuclear transcription factor‐κB (NF‐κB) P38 JNK pathway activation upon binding to TLR4‐MD‐2 through the MyD88 pathway, leading to the subsequent release of TNF‐α,IL‐1, and IL‐6.Immunosuppression mainly occurs 2 days after ischaemic stroke，In the bone marrow, HMGB1 induces proliferation of Myeloid‐derived suppressor cells(MDSCs) and their release to the circulation, and, MDSCs inhibit lymphocyte activation via arginase (ARG1) secretion and might induce lymphocyte apoptosis.

After ischemic cerebral infarction onset, microglia and macrophages in the brain are activated by ischemia and DAMP stimulation, releasing proinflammatory factors such as IL‐1β and TNF‐α (Taylor and Sansing, 2013), which cross the disrupted blood–brain barrier and enter the bloodstream to activate the intrinsic immune system. The aseptic inflammation cascade expands (Jickling et al., 2015) with blood–brain barrier disruption, which exposes the “immune privileged” central nervous system (CNS) to the immune system with the DCs and macrophages that are present as CNS antigens, leading to the activation of the adaptive immune system (Planas et al., 2012). The degree and extent of the inflammatory response are regulated by the CNS and inflammation itself. Ischemic injury to the brain causes an overregulation of the immune system by the CNS, which weakens the immune system. Thus, a local and systemic state of inflammatory response occurs after ischemic brain injury, which is accompanied by systemic immune impairment (Chamorro et al., 2012). HMGB1 can promote microglia activation and IL‐1β and TNF‐α secretion by acting on TLR‐4 on the microglial membrane surface (Yang et al., 2011). Because HMGB1 in the early stages is predominantly present as the reduced‐HMGB1, the level of only disulfide‐HMGB1 with 1000 times higher affinity for TLR‐4 than the reduced‐HMGB1 and oxidized‐HMGB1 increases gradually 24 h after infarction (Liesz et al., 2015), ensuring that only disulfide‐HMGB1 can bind to TLR‐4 and promote cytokine production (Yang et al., 2015). Therefore, HMGB1 release may not be a key mechanism for the initial activation of microglia after infarction. The intracranial HMGB1 injection in different redox states in rats revealed that the disulfide‐HMGB1 can significantly induce the expressions of NF‐κBIα mRNA, Nod‐like receptor 3 (NLRP3) mRNA, and IL‐1b protein 2 h after infarction and the expressions of NF‐κBIα mRNA, TNFα mRNA, and NLRP3 protein 24 h after infarction. However, reduced‐HMGB1 exerts no considerable effect on these inflammatory mediators (Frank et al., 2016), suggesting that HMGB1 may not be a key mediator of intracranial inflammation in the ultra‐early phase of cerebral infarction. Reduced‐HMGB1 forms a heterodimer with CXCL12, which enhances CXCL12 binding to CXCR4 and promotes leucocyte migration (Schiraldi et al., 2012). Additionally, HMGB1 promotes neutrophil migration by binding to RAGE on the neutrophil membrane surface (Orlova et al., 2007). Anti‐HMGB1 neutralizing antibodies significantly reduced the serum IL‐1, TNF‐α, and IL‐6 concentrations in mice 3 days after MCAO (Liesz et al., 2015). The aforementioned findings indicate that HMGB1 is vital for the activation of the peripheral immune system early after cerebral infarction. Early activation of the intrinsic immune system promotes central and peripheral inflammation. Because the blood–brain barrier is disrupted and CNS epitope antigens are exposed to the immune system, monocytes and macrophages, which are responsible for antigen presentation, play a crucial role in mediating the activation of the adaptive immune system. Intracranially annotated disulfide‐HMGB1 not only promotes IL‐1β production but also induces the upregulation of MHC‐II molecule expression in antigen‐presenting cells (Aucott et al., 2018). Moreover, HMGB1 promotes macrophage activation through the RAGE (Muhammad et al., 2008), suggesting that HMGB1 also plays a vital role in the activation of the adaptive immune system after cerebral infarction. Additionally, HMGB1 plays a crucial role in immunosuppression after stroke, which mainly features monocyte hypofunction and lymphocytopenia (Liesz et al., 2009). Three main classical pathways of poststroke immunosuppression exist, namely hypothalamic pituitary adrenal gland axis, sympathetic nervous system, and parasympathetic nervous system. Liesz et al. (2015) reported that blocking of the HMGB1 and RAGE signaling pathways could eliminate cellular immunosuppression and restore lymphocyte function in the subacute phase after stroke. The authors also observed the overactivation of monocytes and depletion of mature monocytes by the HMGB1–RAGE pathway in the acute phase of cerebral infarction. Additionally, the HMGB1/RAGE pathway activation recruits a subpopulation of immature monocytes expressing CD11b^+^ Gr‐1^+^ on the cell membrane surface. The CD11b + Gr‐1^+^ monocyte subpopulation induces apoptosis in activated lymphocytes (Rodríguez and Ochoa, 2008). Additionally, HMGB1/RAGE pathway activation causes expansion of the CD11b +Gr‐1^+^ monocyte subpopulation, which exhibits a hyporesponsive state after stroke, as evidenced by the reduced expressions of MHC‐II and costimulatory molecules and reduced secretion of the proinflammatory cytokines TNF‐α and IL‐12. Poststroke immunosuppression induced by the HMGB1/RAGE pathway is independent of the classical pathway (Liesz et al., 2015). Additionally, HMGB1 promotes apoptosis of T cells by downregulating mitofusin 2 (Mfn2) expression in T cells, thereby activating the mitochondrial apoptotic pathway (Wu et al., 2014). This may also be one of the mechanisms by which HMGB1 promotes poststroke immunosuppression (Figure 4).

### 4 HMGB1 and blood–brain barrier disruption

The blood–brain barrier is composed of an interdependent cell network, which comprises capillary endothelial cells, glial cells, pericytes, and extracellular matrix. An intact blood–brain barrier plays a role in maintaining the relative stability of the intracranial environment (Stokum et al., 2016; Sweeney et al., 2019). Disruption of the blood–brain barrier after cerebral infarction promotes the development of cerebral edema, whereas severe disruption of the blood–brain barrier causes the development of postinfarction hemorrhagic transformation (Stokum et al., 2016). Pathological changes of the blood–brain barrier after brain infarction include swelling of astrocyte end‐feet, detachment of end‐feet from the basement membrane, and disruption of tight junctions between endothelial cells. Zhang et al. (2011) observed that intravenous anti‐HMGB1 monoclonal antibody injection is effective in preventing pathological changes of the blood–brain barrier in a transient MCAO model in mice. MMP9 plays a vital role in disruption of the endothelial cell tight junctions (Piao et al., 2009; Yang et al., 2007). HMGB1/TLR‐4 signaling pathway activation promotes astrocyte‐induced MMP9 secretion, which may be one of the blood–brain barrier disruption mechanisms after cerebral infarction (Qiu et al., 2010).

### 5 HMGB1 and neuronal cell death

The ischemic brain tissue after cerebral infarction can be divided into two regions, namely core and penumbra. The neuronal cells in the core region undergo necrosis at an early stage, whereas those in the penumbra region undergo apoptosis, necrosis, parthanatos, ferroptosis, autolysis, phagoptosis, autophagy, and necroptosis upon simulation due to pathological changes such as ischemia, oxidation, inflammatory mediators, and excitatory amino acids (Kim et al., 2011; Lipton, 1999). Apoptosis occurs in the neurons after the addition of N‐methyl‐D‐aspartic acid (NMDA) to the medium of primary cortical neurons; however, the simultaneous addition of HMGB1 antibody to the medium or transfection with HMGB1 siRNA neurons was shown to prevent NMDA‐induced apoptosis (Kim et al., 2011), which suggests the vital role of HMGB1 in excitatory amino acid‐induced apoptosis. HMGB1 alone does not induce apoptosis in the cortical neurons, suggesting that NMDA induces other substances that act synergistically with HMGB1 to promote apoptosis. Studies have exhibited that HMGB1 promotes neuronal apoptosis through RAGE/extracellular signal‐regulated kinases and RAGE/p38 MAPK pathways involved in excitatory amino acid‐induced neuronal apoptosis (Kim et al., 2011). Autophagy, a cellular “self‐eating” process, is a pathological response to extracellular stimuli that usually plays a role in preventing cell death. However, excessive autophagy can lead to cell death (Klionsky, 2008). The large amount of ROS generated after cerebral infarction promotes HMGB1 transfer by oxidizing the “‐SH” group of cysteine at position 106 in the nucleus, which promotes HMGB1 translocation to the cytoplasm, wherein cytoplasmic HMGB1 binds competitively to beclin1, eventually disrupting the interaction between beclin1 and B‐cell lymphoma‐2 (Bcl‐2) and promoting autophagy (Pan et al., 2020; Tang et al., 2010, 2016). However, the role of HMGB1 in other neuronal death pathways after cerebral infarction has not been reported yet.

### 6 HMGB1 and tissue repair after cerebral infarction

In the late stage of ischemic cerebral infarction, repair processes such as neural and vascular regeneration and synaptic function remodeling occur in the injured brain tissues. Studies have demonstrated the vital role of HMGB1 in the repair of neural tissues after cerebral infarction. HMGB1 released from the activated astrocytes after cerebral infarction promotes EPC proliferation through RAGE, and HMGB1 facilitates EPC migratory aggregation toward the peri‐infarct cortex. Thus, as a signal between astrocytes and EPC, HMGB1 promotes the interaction between astrocytes and EPC, while accelerating postinfarct vascular regeneration and brain tissue repair (Hayakawa et al., 2012). Studies have exhibited that HMGB1 released from the astrocytes promotes EPC proliferation through early growth response‐1 (Egr1) signaling, which further promotes the upregulation of brain endothelial cell RAGE expression, whereas the interaction of endothelial cell RAGE with β2‐integrins on the surface of EPC membranes promotes the adhesion of brain endothelial cells to EPCs and accelerates EPC migration (Hayakawa et al., 2014). Additionally, HMGB1 promotes postinfarction vascular remodeling through many indirect mechanisms. EPC secretion of IL‐8 stimulated by HMGB1 is crucial in postinfarction vascular remodeling (Chen et al., 2014). HMGB1 acting on RAGE on the astrocyte surface causes activation of the downstream PI3K/protein kinase B (AKT) signaling pathway. The HMGB1/IL‐6 signaling pathway is crucial for postinfarction vascular remodeling because anti‐IL‐6 neutralizing antibodies attenuate postinfarction angiogenesis and neurological recovery in mice (Chen et al., 2017). In addition to postinfarction vascular remodeling, HMGB1 is also associated with postinfarction neural regeneration. TLR‐4 and HMGB1 play a vital role in postinfarction neural regenerationby promoting the proliferation and differentiation of neural stem cells through TLR‐4(Moraga et al.,^2014^) . These molecules also facilitate neuroblast migration by promoting the expressions of stromal cell‐derived factor 1 (SDF‐1) and brain‐derived neurotrophic factor (BDNF) (Palma‐Tortosa et al., 2019).

### 7 HMGB1 and subcortical white matter stroke

Subcortical white matter stroke accounts for 25% of stroke incidences and is particularly common in the elderly population, in which it causes severe motor and cognitive dysfunctions (Marin and Carmichael, 2019). Clinical conditions that lead to subcortical white matter stroke include acute focal ischemic white matter lesions caused by deep penetrating arterial occlusions (Caplan, 2015) and chronic diffuse white matter ischemic lesions caused by cerebral small vessel disease (Prins and Scheltens, 2015). Oligodendrocyte glial cell death and myelin deprivation are the typical pathological features of white matter stroke (Rosenzweig and Carmichael, 2013). CNS myelin is mainly composed of oligodendrocytes. Therefore, reducing oligodendrocyte damage under ischemic conditions is the key to treat white matter stroke. In an oligodendrocyte oxygen‐glucose deprivation model, the oligodendrocytes transfected with HMGB1 siRNA exhibited a substantially higher mortality rate than normal oligodendrocytes, which suggests the protective role of HMGB1 in white matter stroke. Another study revealed that the mortality rate of oligodendrocyte transfected with HMGB1 siRNA was significantly higher than that of normal oligodendrocytes in an acute focal white matter stroke mouse model induced by endothelin‐1 (ET‐1) injection. In a mouse endothelin‐1 (ET‐1) injection‐induced acute focal white matter stroke model, HMGB1 released from the nucleus of dead oligodendrocyte cells interacted with TLR2 on the adjacent oligodendrocyte cell membrane in an autocrine manner, thereby promoting downstream ERK1/2 and cAMP‐response element‐binding protein phosphorylation and other prosurvival signaling pathways to attenuate ischemic white matter injury (Choi et al., 2017; Gensel et al., 2015; Martinez et al., 2008). HMGB1 promoted microglial polarization toward M1 phenotype (proinflammatory) through TLR‐4, whereas it promoted microglial polarization toward M2 phenotype (anti‐inflammatory) through TLR‐2 (Gensel et al., 2015; Martinez et al., 2008). Moreover, glycyrrhizin injection did not affect acute focal ET‐1 injection‐induced white matter stroke model, suggesting that HMGB1‐mediated microglia activation may not exacerbate inflammatory injury in acute white matter stroke (Choi et al., 2017). Additionally, the astrocytes release HMGB1 after acute white matter stroke to bind with RAGE on the EPC surface, which promotes EPC aggregation into the injured white matter lesions (Hayakawa et al., 2013). These studies suggest the beneficial role of HMGB1 in acute focal ischemic white matter stroke. Chronic white matter lesions in chronic cerebral ischemia are usually accompanied by long‐term glial cell hyperactivation (Choi et al., 2016), and its downstream TLR4/NF‐κB signaling pathways are continually activated in chronic ischemic white matter lesions. The HMGB1 signaling pathway may play a vital role in the pathological mechanism of chronic ischemic white matter lesions (Hei et al., 2018; Lee et al., 2015). Hei et al. (2019) reported that HMGB1 antibodies inhibit glial cell activation by inhibiting the TLR4/NF‐κB signaling pathway, which attenuates the inflammatory response and causes chronic cerebral ischemia‐induced damage to the optic tract, suggesting that HMGB1 possibly plays a deleterious role in chronic cerebral ischemia‐induced white matter lesions.

### 8 HMGB1 removal

HMGB1 plays a dual role after cerebral infarction by causing both damage and repair. Several studies have exhibited that the blood HMGB1 concentration after cerebral infarction is positively correlated with cerebral infarction severity and poor prognosis (Le et al., 2018; Sapojnikova et al., 2014; Wang et al., 2020). Animal experimental studies have demonstrated that both anti‐HMGB1 antibody and glycyrrhiza sweetener injections can reduce the infarction volume (Chen et al., 2020; Liu et al., 2007; Xiong et al., 2016), indicating that HMGB1 released after cerebral infarction is mainly damaging. Healthy human beings possess the mechanism to remove HMGB1, thereby avoiding HMGB1 from continuously aggravating ischemic brain injury. Among these mechanisms, the transfer of mononuclear macrophages from outside the brain to the infarct site after cerebral infarction by endocytosing HMGB1 is crucial, followed by lysosomal digestion. Studies have exhibited that both macrophage scavenger receptor 1 (MSR1) and macrophage receptor with collagenous structure (MARCO) are involved in HMGB1 endocytosis by mononuclear macrophages, with MSR1 playing a more significant role, whereas the transcription factor v‐maf musculoaponeurotic fibrosarcoma oncogene homolog B (MAFB) is associated with the musculo‐aponeurotic fibrosacroma (Maf) recognition elements (MARE2) in the MSR1 promoter region element (MARE2) site, which promotes MSR1 expression and facilitates endocytic digestion of HMGB1. However, clearance of HMGB1 released after cerebral infarction may be partially dependent on the involvement of MSR1 or MARCO, and further studies are warranted to understand the mechanism of removal of HMGB1 released after cerebral infarction (Shichita et al., 2017).

### 9 Future perspective

HMGB1 is an important molecule released early after ischemic stroke, which plays an important role in the initiation, amplification, and dissipation of inflammation after stroke. Redox modification of HMGB1 plays an important role in this process. Currently, a few studies have explored the redox modification of HMGB1 after ischemic stroke. Studying the effect of different redox modified HMGB1 on ischemic stroke may be an important topic of research. In addition, HMGB1 seems to play different roles in different stroke subtypes, and whether these differences are related to the redox modification of HMGB1 remains unknown. Research in these areas may contribute to a deeper understanding of the pathophysiological mechanism after stroke and provide a theoretical basis for more accurate stroke treatment in the future.

## 4 SUMMARY

HMGB1, which is located in the nucleus under physiological conditions, plays a pivotal role in genetics. When cells are subjected to various stimuli, HMGB1 is modified and translocated to the cytoplasm and in the extracellular space, wherein it plays diverse functional roles. Studies have exhibited the crucial roles of HMGB1 in cell death, immunity and inflammation, thrombosis, and remodeling and repair. HMGB1 released after cerebral infarction is extensively involved in the pathological injury process in the early stage of cerebral infarction, whereas it is involved in the promotion of brain tissue repair and remodeling in the late stage of cerebral infarction. HMGB1 plays a neurotrophic role in acute white matter stroke, whereas it causes sustained activation of inflammation and plays a damaging role in chronic white matter ischemia, indicating that it exhibits diverse functional roles in different cerebral infarction subtypes. Thus, HMGB1 plays a complex role in cerebral infarction, which is related to not only the modification of HMGB1 and bound receptors but also different stages and subtypes of cerebral infarction. The functional roles of HMGB1 in cerebral infarction are poorly understood. Thus, future studies on HMGB1 should investigate the spatial and temporal dynamics of HMGB1 after cerebral infarction. Moreover, future studies on HMGB1 should attempt to integrate different stages and infarct subtypes of cerebral infarction.

## AUTHOR CONTRIBUTIONS

Conceptualization: J.L. and B.G. Investigation: N.H. and H.G. Writing—original draft: B.G. and S.W. Writing—review & editing: G.Z. and M.Z. Validation: J.L., B.G., S.W. Visualization: J.L.

## CONFLICT OF INTERESTSTATEMENT STATEMENT

All other authors declare no competing interests.

## SIGNIFICANCE

The study highlights one of the death‐causing pathological conditions with reference to the DAMP protein HMGB1 that plays crucial roles in the pathophysiology of many inflammation‐related diseases. The scenario is complex as HMGB1 activates several signaling pathways and exhibits different functional features acting as a positive or a negative regulator. In this review, we explain its effect on different subtypes of cerebral infarction and its key mechanism based on the spatiotemporal dynamics and modifications of HMGB1 after cerebral infarction.

### PEER REVIEW

The peer review history for this article is available at https://publons.com/publon/10.1002/brb3.2987.

## Data Availability

The data that support the findings of this study are available from the corresponding author, Mingze Chang, upon reasonable request.
